# Sex differences in response to COVID-19 infection: a retrospective study based on emergency medical team interventions

**DOI:** 10.3389/fpubh.2026.1747666

**Published:** 2026-04-24

**Authors:** Krzysztof Marek Mitura, Daniel Celiński, Olga Bielan, Jadwiga Snarska, Sławomir Dariusz Szajda

**Affiliations:** 1 Independent Public Health Care Center RM-MEDITRANS Emergency Station and Sanitary Transport in Siedlce, Siedlce, Poland; 2 Department of Emergency Medical Service, Medical University of Warsaw, Warsaw, Poland; 3 Department of Nursing, School of Public Health, University of Warmia and Mazury in Olsztyn, Olsztyn, Poland; 4 Department of Surgery, Collegium Medicum, University of Warmia and Mazury in Olsztyn, Olsztyn, Poland; 5 Department of Emergency Medical Service, Collegium Medicum, University of Warmia and Mazury in Olsztyn, Olsztyn, Poland

**Keywords:** COVID-19, emergency medical team, female, male, out-of-hospital emergency medical services, SARS-CoV-2, sex, symptoms

## Abstract

The COVID-19 pandemic adversely affected several aspects of daily life and seriously threatened public health. The main aim of this study was to investigate the differences in human response to SARS-CoV-2 virus infection depending on sex, based on emergency medical team (EMT) interventions. The study was conducted in north-eastern Poland and were based on 12,110 EMT interventions, in which SARS-CoV-2 virus infection was indicated as an ICD-10 codification-based diagnosis. The study used descriptive statistics, Mann–Whitney U, and Chi-square tests. Patients infected with SARS-CoV-2 accounted for 4.6% of all EMT interventions during the period under analysis. Younger males were more likely to acquire infection than females (*p* < 0.001)—the median (Me) age was 68.0 vs. 71.0. In infected males, a life-threatening condition was diagnosed more frequently than in females (*p* < 0.001; 53.6% vs. 48.6%). Females remained at the place of call more often (*p* < 0.001; 46.2% vs. 39.6%), whereas males were more frequently transported to hospital (*p* < 0.001; 59.3% vs. 53.3%) and were more likely to be declared dead (*p* = 0.002; 0.8% vs. 0.4%). In males, the predominant complaints included dyspnoea (*p* < 0.001; 47.2% vs. 38.4%), abnormalities of breathing (*p* = 0.001; 40.1% vs. 37.2%) and cyanosis (*p* < 0.001; 3.2% vs. 2.1%), whereas, in females, these included vomiting (*p* < 0.001; 9.4% vs. 4.4%), diarrhoea (*p* < 0.001; 7.8% vs. 5.0%) and collapse (*p* = 0.034; 8.6% vs. 7.5%). The blood oxygen saturation value (SpO_2_) (*p* < 0.001) in males (Me = 92.0) was significantly lower than that in females (Me = 94.0). Oxygen therapy (*p* < 0.001; 29.0% vs. 24.0%) was more frequently associated with males. The course of the COVID-19 infection depends on the sex of the patient, and male sex is a risk factor for its severe course. Therefore, public health strategies and actions should take into account sex as an important factor in the disease process, prevention and the fight against the pandemic.

## 1 Introduction

The COVID-19 (COrona VIrus Disease 2019) pandemic resulted in numerous negative phenomena affecting everyday life related to social relations, education, mobility, the world economy, and the accessibility and quality of healthcare services (1, 2). The world first heard about COVID-19 at the end of 2019 in the city of Wuhan, China, and in March 2020, the World Health Organization (WHO) declared a COVID-19 pandemic and recommended the application of lockdowns, isolation measures and personal protective equipment. In Poland, an epidemic emergency state was declared on 14 March 2020, and on 20 March 2020, a state of epidemic was announced. The WHO declared the end of the pandemic in May 2023, whereas in Poland, the state of epidemic emergency was annulled on 1 July 2023. Nevertheless, COVID-19 remains a public health concern due to the evolution of SARS-CoV-2 (severe acute respiratory syndrome coronavirus 2), involving, e.g., mutation of the virus to new variants (3, 4).

Apart from having a major impact on everyday life, the COVID-19 pandemic also significantly altered the functioning of healthcare systems worldwide. It brought about changes in the functioning of emergency medical systems (5, 6). Emergency medical teams (EMT), dedicated to providing medical assistance in the event of a health and life emergency, also had to provide services resulting from the emergency caused by the pandemic (7), including to patients suspected of having COVID-19.

The enormous global hazard posed by the COVID-19 pandemic is evidenced by the fact that, according to WHO data, from January 2020 to November 2024 alone, 7.1 million people worldwide, including 121,000 in Poland, died of COVID-19, while 776 million 546 thousand were infected, including 6.7 million people in Poland (8). Despite the effectiveness of COVID-19 vaccines in preventing the disease and reducing the risk of hospitalisation, severe illness and death (9, 10), by the end of 2023, only 67% of the world’s population had completed the full primary series, which typically consists of two doses). Additionally, 32% of individuals had received at least one booster dose. In Poland these figures were 61% for the full primary series and 34% for at least one booster dose (8).

COVID-19 is a complex disease that can lead to multiple serious health complications. The entry point for SARS-CoV-2 is the respiratory tract. Due to the virus’s destructive effects, it can affect several organ systems. Although it can be asymptomatic, it can also cause severe multi-organ failure. COVID-19 can last from 1 to 2 weeks in mild cases and up to 12 weeks in the most severe cases, which is determined by factors such as age, comorbidities and vaccination (11). In the initial phase of the disease, common symptoms include fever, cough, dyspnoea, muscle pain, fatigue, and loss of smell or taste, whereas less common symptoms include headache, haemoptysis and diarrhoea (12, 13). Most people recover from SARS-CoV-2 virus infection, but some experience health problems for weeks, months or even years after infection (14).

The risk factors likely to contribute to the increased incidence of COVID-19 in adults include older age, male sex and ethnic origin, as well as comorbidities, such as cardiovascular diseases, hypertension and chronic obstructive pulmonary disease (15, 16). In contrast, a healthy diet, normal skin condition and vaccination are protective factors against infection and the development of COVID-19 (16).

Sex is a major risk factor affecting both the incidence and severity of viral infections. Data on COVID-19-related mortality indicate that males are more susceptible to the disease than females. Complications such as hypertension, diabetes, kidney diseases and cardiovascular diseases are closely associated with COVID-19 mortality rates. These complications are related to tobacco smoking and alcohol consumption as well as comorbidities, largely associated with the male sex (15).

Global public health policies must consider the impact of the COVID-19 pandemic on sex so that targeted interventions, including prevention and treatment, can be implemented. Collecting data on patients by sex is essential to identify disease factors, risk of ill health, and premature death.

There are several papers on various aspects related to the patient’s sex and its relationship to COVID-19 infection and its course. None of them analyse the issue of patient sex from the EMT perspective. The current study attempts to fill this knowledge gap.

The main aim of this study was to investigate differences in response to the infection in females and males based on pre-hospital medical interventions performed by EMTs in patients diagnosed with SARS-CoV-2 virus infection.

## 2 Materials and methods

The present study focused on Warmińsko-Mazurskie Province located in north-eastern Poland. The area of the province covers 24,173.47 km^2^ (7.7% of Poland’s area), and, at the end of 2023, had a population of 1,357,910 (3.6% of Poland’s population), with females accounting for 51.2% (*n* = 695,285) and males for 48.8% (*n* = 662,625) (17).

The retrospective analysis covered EMT interventions between 1 January 2020, and 31 December 2022. The data used in the study was derived from the State Emergency Medical Service Command Support System (SWD PRM), which is an electronic register of EMT interventions in Poland, and made available by the Warmia and Mazury Provincial Office in Olsztyn, Poland. Of all the EMT interventions in Warmińsko-Mazurskie Province (*n* = 262,738), during which patient’s sex was recorded, 12,110 (4.6%) cases of patients, in whom the main reason for intervention was SARS-CoV-2 virus infection, were identified based on diagnosis groups according to the International Classification of Diseases (ICD-10) (*U00-U85 - Codes for special purposes*). The diagnosis given included: *U04.9 - Severe acute respiratory syndrome [SARS], unspecified* (*n* = 3); *U07.1 - COVID-19, virus identified* (*n* = 10,981); *U07.2 - COVID-19, virus not identified* (*n* = 699)*; U08.9 - Personal history of COVID-19, unspecified* (*n* = 261); *U09.9 - Post COVID-19 condition, unspecified* (*n* = 106) and *U10.9 Multisystem inflammatory syndrome associated with COVID-19, unspecified* (*n* = 60).

The study was conducted with the approval of the Research Ethics Committee of the University of Warmia and Mazury in Olsztyn No. 20/2024, issued on 28 June 2024. The data used in the study was derived from the State Emergency Medical Service Command Support System (SWD PRM), which is an electronic register of EMT interventions in Poland, and made available by the Warmia and Mazury Provincial Office in Olsztyn (Poland). Authors had not access to information that could identify individual participants the study.

The study utilised data including: patient age, sex, viral infection, the area of intervention, the place of call, the intervention outcome, the presence of symptoms, consciousness disturbances, basic vital signs, medical procedures and any additional diagnoses made using the three-character ICD-10 coding system. The analyses only concerned pre-hospital management of patients.

The analysed data were collected in a Microsoft Excel MS Office 2021 database for Windows 11. The study used R ver. 4.3.1 (18) with the RStudio ver. 2023.09.1.494 environment to perform statistical analyses (19).

The normality of the distribution was assessed using the Kolmogorov–Smirnov test. In order to compare the age of the patients (in years) and the examined vital parameters for females and males, the Mann–Whitney non-parametric U test (U) as well as descriptive statistics, particularly the mean (M), median (Me), standard deviation (SD) and interquartile range (IQR), were applied. A Chi-square test (*χ*^2^) was employed to investigate the relationship between qualitative variables, and count (n) and percentage (%) were used to describe them. In addition, when any of the counts of qualitative variables amounted to five or less, the *χ*^2^ test with Yates’ continuity correction was employed. Statistically significant results were recognised at *p* < 0.050.

## 3 Results

From 1 January 2020 to 31 December 2022, EMT interventions due to SARS-CoV-2 virus infection accounted for 4.6% (*n* = 12,110) of all interventions (*n* = 262,738) in which the patient’s sex was specified. Of all the interventions caused by COVID-19 infection, female patients accounted for 51.9% (*n* = 6,283), and male patients for 48.1% (*n* = 5,827).

As for the EMT interventions, significant differences (*p* < 0.001) were noted for the age of females and males infected with SARS-CoV-2 (Table 1). The median age for males was 68 years (M = 65.0), and for females it was 71 (M = 68.6).

**Table 1:** Age of patients diagnosed with SARS-CoV-2 virus infection.

	Parameters	Female	Male	Test	
				*U*	*p*-value
Age (years)	*n*	6,178	5,725	15,090,994	*p* < 0.001
	Mean (SD)	68.6 (18.1)	65.0 (16.8)		
	Median (IQR)	71.0 (60.0–83.0)	68.0 (57.00–77.00)		
	Range	0–102	0–103		

Significant differences (*p* < 0.001) were noted for the number of COVID-19-related EMT interventions and other statistics between females and males (Table 2). Interventions due to SARS-CoV-2 accounted for 4.9% for females (*n* = 6,283) and 4.3% for males (*n* = 5,827).

**Table 2:** Characteristics of interventions in patients diagnosed with SARS-CoV-2 virus infection.

Patient infected with SARS-CoV-2	*N* (%)	Female	Male	Test	
		*n* (%)	*n* (%)	*χ*^2^ (df)	*p*-value
COVID-19 infection					
No	250,628 (95.4)	121,542 (95.1)	129,086 (95.7)	53.070 (1)	p < 0.001
Yes	12,110 (4.6)	6,283 (4.9)	5,827 (4.3)		
Total	262,738 (100)	127,825 (100)	134,913 (100)		
Patient’s age					
0–14	122 (1.0)	63 (1.0)	59 (1.0)	0.00 (1)	*p* = 0.953
15–29	317 (2.7)	176 (2.8)	141 (2.5)	1.71 (1)	*p* = 0.191
30–49	1,517 (12.7)	693 (11.2)	824 (14.4)	26.95 (1)	p < 0.001
50–69	4,074 (34.2)	1,886 (30.5)	2,188 (38.2)	78.07 (1)	p < 0.001
70+	5,873 (49.3)	3,360 (54.4)	2,513 (43.9)	130.85 (1)	p < 0.001
Total	11,903 (100)	6,178 (100)	5,725 (100)		
Area					
Urban	6,625 (54.7)	3,451 (54.9)	2,174 (54.5)	0.25 (1)	*p* = 0.615
Rural	5,485 (45.3)	2,832 (45.1)	2,653 (45.5)		
Total	12,110 (100)	6,283 (100)	4,827 (100)		
Place of call					
Home	11,598 (95.8)	6,053 (96.3)	5,545 (95.2)	10.38 (1)	*p* = 0.001
Public place	401 (3.3)	173 (2.8)	228 (3.9)	12.69 (1)	p < 0.001
Workplace	61 (0.5)	28 (0.4)	33 (0.6)	0.88 (1)	*p* = 0.349
Road traffic	40 (0.3)	21 (0.3)	19 (0.3)	0.01 (1)	*p* = 0.938
School	10 (0.1)	8 (0.1)	2 (0.0)	2.14 (1)**	*p* = 0.143
Total	12,110 (100)	6,283 (100)	5,827 (100)	**χ^2^ test with Yates’ continuity correction	
Outcome of intervention					
Patient did not consent	45 (0.4)	24 (0.4)	21 (0.4)	0.04 (1)	p = 0.845
Patient remained at the place of call	5,212 (43.0)	2,905 (46.2)	2,307 (39.6)	54.44 (1)	p < 0.001
Hospital	6,784 (56.0)	3,331 (53.0)	3,453 (59.3)	9.56 (1)	p < 0.001
Death	69 (0.6)	23 (0.4)	46 (0.8)	9.56 (1)	p = 0.002
Total	12,110 (100)	6,283 (100)	5,827 (100)		
Life-threatening condition					
No	5,935 (49.0)	3,231 (51.4)	2,704 (46.4)	30.48 (1)	p < 0.001
Yes	6,175 (51.0)	3,052 (48.6)	3,123 (53.6)		
Total	12,110 (100)	6,283 (100)	5,827 (100)		

Depending on sex and age, significant differences were observed in patients aged 30 years and older (Table 2). Of all those infected with the virus, depending on sex, the proportion of the infected in each age group was as follows: age group of 30–49 years (*p* < 0.001), 11.2% (*n* = 693) for females, and 14.4% (*n* = 824) for males; age group of 50–69 years (*p* < 0.001), 30.5% (*n* = 1,886) for females, 38.2% (*n* = 2,188) for males; and age group of 70 years and older (*p* < 0.001), 54.4% (*n* = 3,360) for females, and 43.9% (*n* = 2,513) for males.

On the other hand, as regards EMT interventions in urban and rural areas, depending on the sex of the patients (Table 2), no significant differences in the number of infected patients were noted (*p* = 0.615).

Depending on the location of the event, significant differences were noted when comparing EMT interventions in the patient’s home (*p* = 0.001) and public places (*p* < 0.001). For females, the proportion of interventions at home was 96.3% (*n* = 6,053), and 2.8% (*n* = 173) for interventions in public places, whereas for males, these values amounted to 95.2% (*n* = 5,545) and 3.9% (*n* = 228), respectively (Table 2).

Significant differences were also noted in the intervention outcome (Table 2). 46.2% of females diagnosed with SARS-CoV-2 virus infection (*p* < 0.001) (*n* = 2,905), and 39.6% of diagnosed males (*n* = 2,307) remained at the place of call after being provided medical assistance. On the other hand, 59.3% (*n* = 3,453) of infected males and 53.0% (*n* = 3,331) of infected females were transported to hospital. Significant differences were also noted for the completion of EMT interventions due to the death of an infected patient (*p* = 0.002), with the percentage of such interventions accounting for 0.8% (*n* = 46) in males and 0.4% (*n* = 23) in females.

The percentage of EMT interventions during which a life-threatening condition was identified was 53.6% for males (*n* = 3,123) (Table 2) and 48.6% for females (*n* = 3,052), indicating a significant differences between the two groups (*p* < 0.001).

As for respiratory symptoms, significant differences were noted for both dyspnoea (*p* < 0.001) and abnormalities of breathing (*p* = 0.01), depending on the sex of the patient (Table 3). In 47.2% of interventions to males (*n* = 2,660), dyspnoea was observed, whereas in 40.1% of cases, abnormalities in breathing (*n* = 2,335) were noted. For females, dyspnoea was diagnosed in 38.4% (*n* = 2,344) and abnormalities of breathing in 37.2% (*n* = 2,339) cases.

**Table 3:** Respiratory symptoms in patients diagnosed with SARS-CoV-2 virus infection.

Symptom	*N* (%)	Female	Male	Test	
		*n* (%)	*n* (%)	*χ*^2^ (df)	*p*-value
Dyspnoea					
No	6,739 (57.4)	3,763 (61.6)	2,976 (52.8)	93.12 (1)	p < 0.001
Yes	5,004 (42.6)	2,344 (38.4)	2,660 (47.2)		
Total	11,743 (100)	3,997 (100)	5,636 (100)		
Abnormalities of breathing					
No	7,436 (61.4)	3,944 (62.8)	3,492 (59.9)	10.32 (1)	p = 0.001
Yes	4,674 (38.6)	2,339 (37.2)	2,335 (40.1)		
Total	12,110 (100)	6,283 (100)	5,827 (100)		
Respiratory murmurs: rhonchi					
No	11,223 (92.7)	5,848 (93.1)	5,375 (92.2)	3.09 (1)	*p* = 0.079
Yes	887 (7.3)	435 (6.9)	452 (7.8)		
Total	12,110 (100)	6,283 (100)	5,827 (100)		
Respiratory sounds: wheezing					
No	11,400 (94.1)	5,905 (94.0)	5,495 (94.3)	0.56 (1)	*p* = 0.456
Yes	710 (5.9)	378 (6.0)	332 (5.7)		
Total	12,110 (100)	6,283 (100)	5,827 (100)		
Respiratory sounds: crackles					
No	10,609 (87.6)	5,517 (87.8)	5,092 (87.4)	0.48 (1)	*p* = 0.481
Yes	1,501 (12.4)	766 (12.2)	735 (12.6)		
Total	12,110 (100)	6,283 (100)	5,827 (100)		
Respiratory sounds: rales					
No	11,622 (96.0)	6,015 (95.7)	5,607 (96.2)	1.88 (1)	*p* = 0.171
Yes	488 (4.0)	268 (4.3)	220 (3.8)		
Total	12,110 (100)	6,283 (100)	5,827 (100)		
Respiratory sounds: other					
No	11,708 (96.7)	6,097 (97.0)	5,611 (96.3)	5.25 (1)	p = 0.022
Yes	402 (3.3)	186 (3.0)	216 (3.7)		
Total	12,110 (100)	6,283 (100)	5,827 (100)		

Significant differences between females (3.0%, *n* = 186) and males (3.7%, *n* = 216) were noted for respiratory phenomena (Table 3) other (*p* = 0.022) than rhonchi (*p* = 0.079), wheezing (*p* = 0.456), crackles (*p* = 0.481) and rales (*p* = 0.171).

The study of females and males diagnosed with SARS-CoV-2 virus infection noted significant differences in skin symptoms identified in a pre-hospital examination performed by EMT (Table 4). The percentage of abnormalities on skin examination (*p* = 0.026) represented 16.2% (*n* = 943) of cases in males and 14.7% (*n* = 925) of cases in females. On the other hand, cyanosis (*p* < 0.001) was present in 3.2% (*n* = 174) of males and 2.1% (*n* = 126) of females infected with SARS-CoV-2. Differences were also noted in the occurrence of peripheral (p < 0.001) and central cyanosis (*p* = 0.005), with 1.4% (*n* = 86) and 0.5% (*n* = 30) for females, and 2.2% (*n* = 131) and 0.9% (*n* = 52) for males, respectively. Dry skin (*p* = 0.035) was found in 11.0% (*n* = 685) of infected females and 9.9% of males (*n* = 565), whereas moist skin (*p* < 0.001) was found in 5.4% (*n* = 334) of females and 7.7% (*n* = 444) of males. Differences in the body temperatures of patients were also shown. Body temperature within normal (*p* < 0.001) was noted for 53.2% (*n* = 3,264) cases in females and 49.2% (*n* = 2,791) cases in males, whereas temperature above normal (n < 0.001) was noted for 49.4% of males (*n* = 2,806), and 45.7% (*n* = 2,806) of females. In contrast, no differences were observed in skin symptoms such as pallor (*p* = 0.552), erythema (*p* = 6.86) and yellowing (*p* = 0.567).

**Table 4:** Cyanosis and skin symptoms in patients diagnosed with SARS-CoV-2 virus infection.

Symptom	*N* (%)	Female	Male	Test	
		*n* (%)	*n* (%)	*χ*^2^ (df)	*p*-value
Cyanosis					
No	11,084 (97.4)	5,798 (97.9)	5,286 (96.8)	12.44 (1)	p < 0.001
Yes	300 (2.6)	126 (2.1)	174 (3.2)		
Total	11,384 (100)	5,924 (100)	5,460 (100)		
Peripheral cyanosis					
No	11,893 (98.2)	6,197 (98.6)	5,696 (97.8)	13.29 (1)	p < 0.001
Yes	217 (1.8)	86 (1.4)	131 (2.2)		
Total	12,110 (100)	6,283 (100)	5,827 (100)		
Central cyanosis					
No	12,028 (99.3)	6,253 (99.5)	5,775 (99.1)	7.74 (1)	p = 0.005
Yes	82 (0.7)	30 (0.5)	52 (0.9)		
Total	12,110 (100)	6,283 (100)	5,827 (100)		
Skin in normal condition					
No	1,868 (15.4)	925 (14.7)	943 (16.2)	4.94 (1)	p = 0.026
Yes	10,242 (84.6)	5,358 (85.3)	4,884 (83.8)		
Total	12,110 (100)	6,283 (100)	5,827 (100)		
Pale skin					
No	10,545 (87.1)	5,482 (87.3)	5,063 (86.9)	0.35 (1)	*p* = 0.552
Yes	1,565 (12.9)	801 (12.7)	764 (13.1)		
Total	12,110 (100)	6,283 (100)	5,827 (100)		
Erythematous skin					
No	11,996 (99.1)	6,226 (99.1)	5,770 (99.0)	0.16 (1)	*p* = 0.686
Yes	114 (0.9)	57 (0.9)	57 (1.0)		
Total	12,110 (100)	6,283 (100)	5,827 (100)		
Yellowed skin					
No	12,080 (99.8)	6,269 (99.8)	5,811 (99.7)	0.33 (1)	*p* = 0.567
Yes	30 (0.2)	14 (0.2)	16 (0.3)		
Total	12,110 (100)	6,283 (100)	5,827 (100)		
Skin moisture					
Normal	9,908 (83.0)	5,185 (83.6)	4,723 (82.4)	2.93 (1)	*p* = 0.087
Dry	1,250 (10.5)	685 (11.0)	565 (9.9)	4.46 (1)	p = 0.035
Moist	778 (6.5)	334 (5.4)	444 (7.7)	27.29 (1)	p < 0.001
Total	11,936 (100)	6,204 (100)	5,732 (100)		
Skin temperature					
Cool	149 (1.3)	68 (1.1)	81 (1.4)	2.41 (1)	*p* = 0.121
Warm	5,612 (47.5)	2,806 (45.7)	2,806 (49.4)	16.22 (1)	p < 0.001
Normal	6,055 (51.2)	3,264 (53.2)	2,791 (49.2)	19.10 (1)	p < 0.001
Total	11,816 (100)	6,138 (100)	5,678 (100)		

No significant differences were noted for pulse irregularity (*p* = 0.193), psychomotor agitation (*p* = 0.910), and the individual Glasgow Coma Scale (GCS) ranges (Table 5). In contrast, significant differences were observed for vomiting (*p* < 0.001), which accounted for 9.4% (*n* = 518) in females and 4.4% (*n* = 240) in males. On the other hand, diarrhoea (*p* < 0.001) and collapse (*p* = 0.034) in females accounted for 7.8% (*n* = 467) and 8.6% (*n* = 511), and in males for 5.0% (*n* = 275) and 7.5% (*n* = 411) cases, respectively. Significant differences were also observed for the percentage of the occurrence of abdominal pain on palpation (*p* < 0.001) between females (3.9%, *n* = 243) and males (3.2%, *n* = 385), and alcohol breath (*p* < 0.001), the percentage of which accounted for 0.2% (*n* = 10) and 0.6% (*n* = 33) of cases, in females and males, respectively. The percentage of patients with psychomotor test scores described as being normal (*p* = 0.011) was observed in 73.6% (*n* = 4,421) of females and 75.6% (*n* = 4,199) of males, whereas sluggishness (*p* = 0.011) was noted for 25.0% (*n* = 1,502) of females and 23.0% (*n* = 1,275) of males. Detailed data are presented in Table 5.

**Table 5:** Selected symptoms in patients diagnosed with SARS-CoV-2 virus infection.

Symptom	*N* (%)	Female	Male	Test	
		*n* (%)	*n* (%)	*χ*^2^ (df)	*p*-value
Pulse					
Regular	10,242 (91.4)	5,296 (91.1)	4,946 (91.8)	1.69 (1)	*p* = 0.193
Irregular	961 (8.6)	518 (8.9)	443 (8.2)		
Total	11,203 (100)	5,814 (100)	5,389 (100)		
Vomiting					
No	10,679 (93.0)	5,420 (90.6)	5,259 (95.6)	110.47 (1)	p < 0.001
Yes	800 (7.0)	560 (9.4)	240 (4.4)		
Total	11,479 (100)	5,980 (100)	5,499 (100)		
Diarrhoea					
No	10,733 (93.5)	5,512 (92.2)	5,221 (95.0)	37.31 (1)	p < 0.001
Yes	742 (6.5)	467 (7.8)	275 (5.0)		
Total	11,475 (100)	5,979 (100)	5,496 (100)		
Collapse					
No	10,517 (91.9)	5,446 (91.4)	5,071 (92.5)	4.50 (1)	p = 0.034
Yes	922 (8.1)	511 (8.6)	411 (7.5)		
Total	11,439 (100)	5,957 (100)	5,482 (100)		
Abdominal pain on palpation					
No	11,725 (96.8)	6,040 (96.1)	5,686 (97.6)	20.10 (1)	p < 0.001
Yes	385 (3.2)	243 (3.9)	142 (2.4)		
Total	12,110 (100)	6,283 (100)	5,827 (100)		
Alcohol breath					
No	12,067 (99.6)	6,273 (99.8)	5,794 (99.4)	14 (14.17)	p < 0.001
Yes	43 (0.4)	10 (0.2)	33 (0.6)		
Total	12,110 (100)	6,283 (100)	5,827 (100)		
Psychomotor test score					
Normal	8,620 (74.5)	4,421 (73.6)	4,199 (75.6)	6.43 (1)	p = 0.011
Agitated	166 (1.4)	87 (1.4)	79 (1.4)	0.01 (1)	*p* = 0.910
Sluggish	2,777 (24.0)	1,502 (25.0)	1,275 (23.0)	6.52 (1)	p = 0.011
Total	11,563 (100)	6,010 (100)	5,553 (100)		
Glasgow coma scale (score range)					
15	10,536 (88.4)	5,456 (88.2)	5,083 (88.7)	0.90 (1)	*p* = 0.342
14–9	1,166 (9.8)	630 (10.2)	536 (9.4)	2.32 (1)	*p* = 0.128
8–3	214 (1.8)	103 (1.7)	111 (1.9)	1.25 (1)	*p* = 0.264
Total	11,916 (100)	6,189 (100)	5,730 (100)		

Significant differences were also noted for some vital signs measured in females and males diagnosed with SARS-CoV-2 virus infection (Table 6). These differences concerned the respiratory rate (*p* < 0.001). The median for both sexes was 18.0; however, the average value for females was 17.9, and for males, it was 18.3 breaths per minute. Differences were observed for the SpO_2_ value (*p* < 0.001), with the median 94.0% for females and 92.0% in males. On the other hand, the median heart rate (*p* < 0.001) in females infected with the virus was noted at a level of 88.0, whereas in males, at 90.0 beats per minute. In addition, the median glycaemic value (*p* = 0.037) for females was 137.0, whereas for males, it was 139.0 mg%. Differences were also noted for the pain scale (*p* < 0.001), with the median for both sexes amounting to 0.0 on the Numerical Rating Scale (NRS). However, the mean score for females was 0.8, whereas for males, it was 0.6. As regards mean arterial pressure (*p* = 0.876), systolic pressure (*p* = 0.195) and diastolic pressure (*p* = 0.338) and the degree of consciousness disturbances evaluated according to the GCS scale (*p* = 0.369), no significant differences were noted.

**Table 6:** Basic vital signs in patients diagnosed with SARS-CoV-2 virus infection.

Vital signs	Parameters	Female	Male	Test	
				*U*	*p*-value
Respiratory rate (per minute)	*n*	6,049	5,601	15,994,518	p < 0.001
	Mean (SD)	17.9 (4.1)	18.3 (4.5)		
	Median (IQR)	18.0 (16.0–18.0)	18.0 (16.0–18.0)		
	Range	8–45	16–50		
Blood oxygen saturation value – SpO_2_ (%)	*n*	6,119	5,634	14,945,104	p < 0.001
	Mean (SD)	90.9 (8.7)	89.3 (9.2)		
	Median (IQR)	94.0 (88.0–97.0)	92.0 (86.0–96.0)		
	Range	50–100	50–100		
Mean arterial pressure (mmHg)	*n*	5,946	5,428	16,110,227	*p* = 0.876
	Mean (SD)	94.6 (14.5)	94.4 (14.3)		
	Median (IQR)	95.0 (85.4–103.5)	95.0 (86.2–102.5)		
	Range	36.0–173.0	30.5–163.0		
Arterial systolic blood pressure (mmHg)	*n*	5,946	5,428	15,911,661	*p* = 0.195
	Mean (SD)	133.4 (23.2)	132.3		
	Median (IQR)	130.0 (120.0–149.0)	130.0 (120.0–145.0)		
	Range	50.0–260.0	50.0–250.0		
Arterial diastolic blood pressure (mmHg)	*n*	5,946	5,428	15,972,528	*p* = 0.338
	Mean (SD)	77.9 (12.8)	78.2 (12.7)		
	Median (IQR)	80.0 (70.0–85.0)	80.0 (70.0–85.0)		
	Range	20.0–154.0	20.0–140.0		
Pulse (per minute)	*n*	6,172	5,682	16,314,214	p < 0.001
	Mean (SD)	89.8 (17.2)	91.7 (17.6)		
	Median (IQR)	88.0 (80.0–100.0)	90.0 (80.0–100.0)		
	Range	36–205	32–220		
Glycaemic value (mg%)	*n*	3,733	3,272	5,931,142	p = 0.037
	Mean (SD)	156.4 (71.1)	160.4 (74.5)		
	Median (IQR)	137.0 (116.0–172.0)	139.0 (117.0–175.0)		
	Range	30–660	35.0–628.0		
Numerical rating scale (points)	*n*	5,400	5,013	12,655,075	p < 0.001
	Mean (SD)	0.8 (1.7)	0.6 (1.5)		
	Median (IQR)	0.0 (0.0–0.0)	0.0 (0.0–0.0)		
	Range	0–10	0–10		
Glasgow coma scale (points)	*n*	6,186	5,730	17,629,237	*p* = 0.369
	Mean (SD)	14.6 (1.5)	14.6 (1.7)		
	Median (IQR)	15.0 (15.0–15.0)	15.0 (15.0–15.0)		
	Range	3–15	3–15		

Significant differences between females and males were also noted for the medical procedures under analysis (Table 7). The percentage of interventions during which an antigen test for the virus was performed (*p* = 0.010) accounted for 28.3% (*n* = 1,778) in females and 26.2% (*n* = 1,578) in males. Oxygen therapy (*p* < 0.001) was applied in 24.0% (*n* = 1,510) of cases in females, and in 29.0% (*n* = 1,689) of cases in males, the intravenous access (*p* = 0.002) was performed for 44.8% (*n* = 2,815) of females and 47.6% (*n* = 2,772) of males, and electrocardiography (*p* = 0.026) was carried out on 29.1% (*n* = 1,831) of females, and 27.3% (*n* = 1,592) of males.

**Table 7:** Selected medical procedures performed on patients diagnosed with SARS-CoV-2 virus infection.

Medical procedure	*N* (%)	Female	Male	Test	
		*n* (%)	*n* (%)	*χ*^2^ (df)	*p*-value
Antigen test					
No	8,805 (72.7)	4,505 (71.7)	4,300 (73.8)	6.67 (1)	*p* = 0.010
Yes	3,305 (27.3)	1,778 (28.3)	1,527 (26.2)		
Total	12,110 (100)	6,283 (100)	5,827 (100)		
Oxygen therapy					
No	8,911 (73.6)	4,773 (76.0)	4,138 (71.0)	38.15 (1)	*p* < 0.001
Yes	3,199 (26.4)	1,510 (24.0)	1,689 (29.0)		
Total	12,110 (100)	6,283 (100)	5,827 (100)		
Electrocardiography					
No	8,687 (71.7)	4,452 (70.9)	4,235 (72.7)	4.94 (1)	*p* = 0.026
Yes	3,423 (28.3)	1,831 (29.1)	1,592 (27.3)		
Total	12,110 (100)	6,283 (100)	5,827 (100)		
Intravenous access					
No	6,523 (53.9)	3,468 (55.2)	3,055 (52.4)	9.32 (1)	*p* = 0.002
Yes	5,587 (46.1)	2,815 (44.8)	2,772 (47.6)		
Total	12,110 (100)	6,283 (100)	5,827 (100)		

There were significant differences between the female and male groups in the diagnoses given after the indication of SARS-CoV-2 virus infection as the primary diagnosis (Table 8). These differences concerned the percentage of diagnoses groups including endocrine and metabolic diseases (*p* = 0.014; 3.6% for females, *n* = 170; 2.7% for males, *n* = 115), and neoplasms (*p* = 0.033; 0.4% for females, *n* = 21; 0.8% for males, *n* = 34).

**Table 8:** ICD-10 groups – second diagnosis given to patients diagnosed with SARS-CoV-2 virus infection.

Diagnosis group (group ICD-10)	*N* (%)	Female	Male	Test	
		*n* (%)	*n* (%)	*χ*^2^ (df)	*p*-value
Symptoms, signs and abnormal clinical findings (R00–R99)	6,626 (73.9)	3,444 (73.2)	3,182 (74.7)	2.38 (1)	*p* = 0.123
Diseases of the circulatory system (I00–I99)	737 (8.2)	382 (8.1)	355 (8.3)	0.13 (1)	*p* = 0.721
Diseases of the respiratory system (J00–J99)	470 (5.2)	245 (5.2)	225 (5.3)	0.02 (1)	*p* = 0.882
Endocrine, nutritional and metabolic diseases (E00–E90)	285 (3.2)	170 (3.6)	115 (2.7)	6.10 (1)	p = 0.014
Injury, poisoning and certain consequences of external causes (S00–T98)	180 (2.0)	99 (2.1)	81 (1.9)	0.48 (1)	*p* = 0.491
Factors influencing health status and contact with health services (Z00–Z99)	174 (1.9)	92 (2.0)	82 (1.9)	0.01 (1)	*p* = 0.912
Diseases of the digestive system (K00–K93)	115 (1.3)	69 (1.5)	46 (1.1)	2.66 (1)	*p* = 0.103
Mental and behavioural disorders (F00–F99)	88 (1.0)	50 (1.1)	38 (0.9)	0.68 (1)	*p* = 0.411
Neoplasms (C00–D48)	55 (0.6)	21 (0.4)	34 (0.8)	4.52 (1)	p = 0.033
Diseases of the nervous system (G00–G99)	48 (0.5)	24 (0.5)	24 (0.6)	0.12 (1)	*p* = 0.732
Other	185 (2.1)	106 (2.3)	79 (1.9)	1.77 (1)	*p* = 0.183
Total	8,963 (100)	4,702 (100)	4,261 (100)		

Significant differences between the sexes were also noted for the 10 most frequent diagnoses given as a subsequent (second) diagnosis (Table 9). At the same time, it can be observed that the most common second diagnoses indicate the main symptoms rather than a specific disease entity. The percentage of diagnoses related to breathing abnormalities (*p* < 0.001) in females amounted to 23.3% (*n* = 1,095), and in males to 30.4% (*n* = 1,296). Similarly, significant differences were noted for *f*ever (*p* = 0.023; 15.2% for females, *n* = 714; 16.9% for males, *n* = 722); malaise and fatigue (*p* = 0.004; 11.8% for females, *n* = 556; 9.9% for males, *n* = 423); arterial hypertension (*p* = 0.020; 3.2% for females, *n* = 152; 2.4% for males, *n* = 103); abdominal pain (*p* < 0.001; 3.5% for females, *n* = 166; 1.9% for males, *n* = 81); nausea and vomiting (*p* < 0.001; 3.8% for females, *n* = 177; 1.6% for males, *n* = 67); and atrial fibrillation (*p* = 0.047; 2.2% for females, *n* = 102; 1.6% for males, *n* = 68).

**Table 9:** Second ICD-10 diagnosis given to patients diagnosed with SARS-CoV-2 virus infection.

Diagnosis (ICD-10 code)	*N* (%)	Female	Male	Test	
		*n* (%)	*n* (%)	*χ*^2^ (df)	*p*-value
Abnormalities of breathing (R06)	2,391 (26.7)	1,095 (23.3)	1,296 (30.4)	58.05 (1)	p < 0.001
Fever of other and unknown origin (R50)	1,436 (16.0)	714 (15.2)	722 (16.9)	5.14 (1)	p = 0.023
Malaise and fatigue (R53)	979 (10.9)	556 (11.8)	423 (9.9)	8.27 (1)	p = 0.004
Syncope and collapse (R55)	373 (4.2)	212 (4.5)	161 (3.8)	2.99 (1)	*p* = 0.084
Essential (primary) hypertension (I10)	255 (2.8)	152 (3.2)	103 (2.4)	5.38 (1)	p = 0.020
Abdominal and pelvic pain (R10)	247 (2.8)	166 (3.5)	81 (1.9)	22.15 (1)	p < 0.001
Nausea and vomiting (R11)	244 (2.7)	177 (3.8)	67 (1.6)	40.56 (1)	p < 0.001
Cough (R05)	222 (2.5)	108 (2.3)	114 (2.7)	1.33 (1)	*p* = 0.250
Pain in throat and chest (R07)	218 (2.4)	111 (2.4)	107 (2.5)	0.21 (1)	*p* = 0.644
Atrial fibrillation and flutter (I48)	170 (1.9)	102 (2.2)	68 (1.6)	3.95 (1)	p = 0.047
Other	2,428 (27.1)	1,309 (27.8)	1,119 (26.3)	2.82 (1)	*p* = 0.093
Total	8,963 (100)	4,702 (100)	4,261 (100)		

## 4 Discussion

The pandemic triggered by the SARS-CoV-2 virus was a global challenge to public health. At the same time, it significantly affected multiple dimensions of social and economic life, contributing to far-reaching transformations in the organization, accessibility, and overall functioning of healthcare systems (2). The rate of its spread and the initial difficulties in diagnosing infected people posed a challenge to the healthcare system. However, as reported by a Polish study, the response of medical services during the pandemic were rated positively by patients despite considerable delays in providing healthcare services (20). Numerous symptoms accompanying the COVID-19 infection were determined by multiple factors, with the sex of the patient being one of them (15, 16). A literature review revealed a lack of studies analysing the accompanying symptoms of COVID-19 patients in males and females based on EMT interventions. Therefore, the authors decided to investigate differences in the body’s response to SARS-CoV-2 virus infection in females and males based on pre-hospital medical interventions carried out by EMTs. Understanding the differences in the response to SARS-CoV-2 infection between sexes can help EMTs better understand disease progression and improve diagnosis. The sudden increase in calls for EMTs due to suspected infections can predict increases in COVID-19 cases and forecast hospital bed usage 1–2 weeks in advance (21).

During the period under analysis, for every 22 interventions, one (4.6%) involved a patient with COVID-19, with infected female patients accounting for 51.9% and infected male patients accounting for 48.1%. At the same time, EMT interventions related to SARS-CoV-2 were significantly more frequent for females than males, accounting for 4.9 and 4.3% of all EMT interventions (Table 2). A study involving patients admitted to a Wuhan (China) hospital during the initial pandemic phase indicated that 73% of the infected were males (12). On the other hand, a meta-analysis of 59 studies involving 36,470 patients showed that, statistically, the risk of COVID-19 was 8% higher for males than for females (22).

The present study shows that more younger men (Me = 68.0, M = 65.0) acquired SARS-CoV-2 infection than females (Me = 71.0, M = 68.6) (Table 1). Up to the age of 30 years, no differences between sexes were noted in infection rates. In the age groups of 30–49 years (14.4% vs. 11.2%) and 50–69 years (38.2% vs. 30.5%), males were predominant among the infected, whereas females were predominant in the age group of 70 years and above (54.4% vs. 43.9%). This may be because testosterone makes males more susceptible to COVID-19 infections, whereas oestrogen serves a protective role in females (23, 24). At the same time, females differ from males in their response to viral infections because they have a stronger inflammatory and humoral immune response, resulting in a lower incidence and intensity of viral infections in females (25). However, in patients over 70 years of age, particularly in older females, the observed decline in immune system function and the high prevalence of comorbidities significantly increase the risk of infection (22, 23). This is confirmed by Zaher et al. (15), who observed that patients with COVID-19 over 60 years of age were mainly females (57%).

Our research indicates a lack of significant differences noted for the number of people infected in urban and rural areas depending on the patient’s sex (Table 2), which may indicate that the virus spreads independently of the area of residence. The predominant EMT interventions were carried out in the patient’s home, which accounted for 96.3% of interventions for females and 95.2% for males. On the other hand, in interventions in public places, infected males accounted for 3.9%, and infected females for 2.8% (Table 2). These differences are probably due to the increased mobility of males compared to females, whereas the predominant proportion of interventions in the patient’s home resulted from the introduced lockdowns and restrictions on movement.

Based on the authors’ own research, it was observed that EMT leaders, based on a physical examination, observed symptoms and vital signs in males, identified a life-threatening condition more frequently (53.6%) than in females (48.6%) (Table 4). The present study indicates that after being provided with medical assistance, females diagnosed with SARS-CoV-2 infection were more likely to remain at the place of call than males (46.2% vs. 39.6%), whereas males (59.3% vs. 53.3%) were transported to hospital more often than females (Table 2). Significant differences were also noted regarding actions following the death of the infected patient, particularly among males. Such interventions accounted for 0.8%, whereas for females, it was 0.4% (Table 2). This may have been due to a more severe course of infection in males than in females, in which female and male sex hormones and the immune response to the infection play an important role. In addition, males are more susceptible to many infectious diseases and show a higher associated mortality rate than females (26). Infectious respiratory diseases tend to be more severe in males than in females, with a higher mortality rate observed simultaneously (22). Accordingly, as reported by Amgalan et al. (27) and Green et al. (28), men were more likely than women to experience a severe course of COVID-19. This was confirmed by a study by Ciarambino et al. (23) who pointed out that males are twice as likely as females to acquire severe fatal SARS-CoV-2, with a mortality rate in COVID-19 patients amounting to 2.8% for females, and 4.7% for males. Similarly, Green et al. (28) observed that, regardless of age, mortality rates were higher in infected men than in women. On the other hand, Pijls et al. (22) demonstrated that males exhibit not only a higher risk of acquiring COVID-19 infection but also a higher risk of a severe course of the disease (by 18%) and a higher number of deaths and ICU admissions compared to females. A Korean study (29) showed that 0.5% of infected patients died of COVID-19, with 59.1% of patients being male.

The present study suggests that for COVID-19 patients, dyspnoea (47.2% vs. 38.4%) and abnormalities of breathing (40.1% vs. 37.2%) were predominant in males (Table 3). Similarly, cyanosis was diagnosed more frequently in males (Table 4) than in females (3.2% vs. 2.1%), both peripheral (2.2% vs. 1.4%) and central (0.9% vs. 0.5%). At the same time, the respiratory rate (Table 6) in males was higher than that in females, with a simultaneous lower SpO_2_ value (Me = 92.0 vs. Me = 94.0). In male COVID-19 patients (Table 4), the following symptoms were more prevalent compared to female patients: body temperature above normal (49.4% vs. 45.7%), skin abnormalities (16.2% vs. 14.7%), and moist skin (7.7% vs. 5.4%). Infected males also exhibited (Table 6) a faster heart rate (Me = 90.0 vs. Me = 88.0) and higher glycaemic levels (Me = 139.0 vs. Me = 137.0). The present study shows (Table 7) that oxygen therapy was applied more frequently in males (29.0% vs. 24.0%), as intravenous access was established in males more frequently (47.6% vs. 44.8%) than in females (Table 7). In addition, it was observed that alcohol consumption was more frequent for virus-infected males than for females (0.6% vs. 0.2%) (Table 5). Moreover, in males infected with SARS-CoV-2, the study found a higher incidence of neoplasms (0.8% vs. 0.4%), as subsequent (second) diagnoses (Table 8). However, based on the 10 most frequent diagnoses, which in most cases indicate the main symptoms rather than a specific disease entity (Table 9), both abnormalities of breathing (30.4% vs. 23.3%) and fever (16.9% vs. 15.2%) were observed more frequently in males than in females. All of the analysed symptoms, vital signs, diagnoses and performed medical procedures indicate that infected males, compared to females, were more likely to have respiratory disorders with concomitant abnormal respiratory parameters. Thus, the current observations are confirmed by a study by Zaher et al. (15), according to which COVID-19 respiratory symptoms predominantly affect the male sex. Moreover, Pimlott et al. (30) reported that the occurrence of symptoms, such as dyspnoea (40.9% vs. 27.5%) and fever (40.9% vs. 29.4%), was more frequently associated with males. On the other hand, a meta-analysis by de Aguiar et al. (31) showed that 29% of patients with COVID-19 had skin symptoms, with inflammatory symptoms classified as rashes accounting for 63%, lesions of vascular origin for 9%, and frostbite-like lesions for 5% of cases. A meta-analysis also showed no statistically significant association between the patient’s sex and symptoms and no significant difference between the severity of COVID-19 infection and skin symptoms in males and females. Numerous clinical data indicate that the comorbidities more commonly affecting males, including chronic obstructive pulmonary disease, obesity, diabetes, cerebrovascular disease, neoplasms and hypertension, increase the risk of severe COVID-19 infection and are associated with poorer treatment outcomes (15, 25).

Based on the present study, it can be concluded that infected females were more likely to suffer from gastrointestinal disturbances than infected males (Table 5). The study noted a more frequent occurrence in females of vomiting (9.4% vs. 4.4%), diarrhoea (7.8% vs. 5.0%) and abdominal pain found on palpation (3.9% vs. 3.2%). In addition, collapse (8.6% vs. 7.5%), psychomotor sluggishness (25.0% vs. 23.0%), and the occurrence of dry skin (11.0% vs. 9.9%) more often affected the female sex (Tables 4, 5) than the male sex. Moreover, based on the (NRS) pain scale values, the study noted differences regarding the severity of pain (Table 6), which were higher in females (M = 0.8 vs. M = 0.6) than in males. As for medical procedures, antigen tests for the virus and electrocardiography were performed more frequently on females (Table 7) than males (28.3% vs. 26.2 and 29.1% vs. 27.3%, respectively). Based on own observations, the authors of this study found a higher prevalence of endocrine and metabolic diseases (3.6% vs. 2.7%) among women infected with SARS-CoV-2 (Table 8). The current study, based on the 10 most frequent second diagnoses (Table 9), found that the following symptoms occur more frequently in females than in males: malaise and fatigue (11.8% vs. 9.9%), arterial hypertension (3.2% vs. 2.4%), abdominal pain (3.5% vs. 1.9%), nausea and vomiting (3.8% vs. 1.6%) as well as atrial fibrillation (2.2% vs. 1.6%). Moreover, Raimondi et al. (32) noted that gastrointestinal symptoms, including nausea, vomiting and diarrhoea, were significantly more prevalent in females (24.6% vs. 15.7%) than in males. The observed higher prevalence of several additional diagnoses in females, beyond hypertension and atrial fibrillation, was related to symptomatic diagnoses closely associated with COVID-19-accompanying symptoms. This may be because females tend to report more severe pain and physical complaints and more numerous, intense and frequent somatic symptoms than males (33).

Sex is therefore a determining factor in the incidence, symptomatology, severity, and mortality associated with SARS-CoV-2 infection. The observed differences between males and females in COVID-19 outcomes may be attributed to immune responses, genetic and hormonal differences, expression of the angiotensin converting enzyme 2 (ACE-2), as well as differences in behavior and lifestyle (27, 28, 34, 35).

The current study is based on the data acquired from the nationwide, integrated State Emergency Medical Service Command Support System, which is undoubtedly the study’s major advantage. It must be considered, however, that the population under study comprised patients who were given pre-hospital diagnoses, the accuracy of which was not checked, yet the symptoms were recorded, and many of the measurable parameters may serve as a tool for their verification. In addition, it should be noted that the analysed data have moderate deficiencies in demographics, symptoms, and vital signs of the population under study due to the specific nature of EMT work. A further limitation of this study is the lack of data regarding COVID-19 vaccination status. This is due to the structure of the medical documentation maintained by emergency medical teams, in which such information was not routinely recorded. Consequently, the impact of vaccination on the clinical course and symptomatology of COVID-19 could not be assessed.

## 5 Conclusion

The patient’s sex determines the course of COVID-19 infection, and the differences in symptomatology between males and females may provide a basis for a better understanding of the different courses of the disease in patients of both sexes. Regarding the symptoms of SARS-CoV-2 infection, breathing abnormalities affect males more frequently, whereas in females, gastrointestinal disturbances are predominant. The male sex is a risk factor for a severe course of infection.

Identifying differences in symptoms between the sexes in response to the infection can help make appropriate medical decisions. The therapeutic process in patients diagnosed with COVID-19 should be based on determinants relating to the patient’s sex and the resulting potential risks. Public health and health policy strategies must consider sex as a key factor influencing the disease process, prevention and pandemic responses.

## Data Availability

The raw data supporting the conclusions of this article will be made available by the authors, without undue reservation.
